# Infection-associated platelet dysfunction of canine platelets detected in a flow chamber model

**DOI:** 10.1186/1746-6148-9-112

**Published:** 2013-06-07

**Authors:** Annika Ferkau, Hans-Jörg Gillmann, Reinhard Mischke, Simone Calmer, Silke Ecklebe, Monia Abid, Jan-Wighard Minde, Frank Echtermeyer, Gregor Theilmeier

**Affiliations:** 1 Department of Anesthesiology and Intensive Care Medicine, Hannover Medical School, Carl-Neuberg-Straße 1, Hannover D-30625, Germany; 2 Small Animal Clinic, Hannover School of Veterinary Medicine, Bünteweg 9, Hannover D-30559, Germany

**Keywords:** Dog, Flow chamber, Inflammation, PAR 4 agonist

## Abstract

**Background:**

In the present study, the influence of bacterial infection, lipopolysacharides (LPS) and hydroxyethyl starch (HES) on platelet function in a parallel plate flow chamber were measured. Experiments were performed with non-activated and protease-activating-receptor (PAR) 4 agonist activated platelets. Comparative measurements were in vivo capillary bleeding time, platelet function analyzer and impedance aggregometry.

**Results:**

PAR 4 agonist did not increase platelet adhesion of platelets from dogs with bacterial inflammation in the flow chamber in contrast to platelets of healthy dogs. Except from impedance aggregometry with lower sensitivity and specificity, PFA did not detect platelet dysfunctions in dogs with infection. In vitro addition of LPS or HES significantly reduced platelet covered area after PAR-activation.

**Conclusions:**

The flow chamber detects platelet dysfunctions in dogs with inflammatory diseases. In vitro addition of LPS highlights the inhibiting effect of bacterial wall components on platelet function. Platelet dysfunction induced by infection could possibly also be diagnosed after treatment of sepsis with colloids has commenced. The flow chamber could be a useful tool to detect sepsis associated platelet dysfunction given that larger prospective trials confirm these findings from a proof of concept study.

## Background

Inflammation leads to activation of coagulation. In addition, an exaggerated coagulability results in a further augmentation of inflammation [1-3]. This mechanism is based on the expression of tissue factor by mononuclear- and endothelial cells, activated by pro-inflammatory cytokines like interleukin 6 [4,5]. Furthermore, important natural occurring anticoagulant pathways including antithrombin, the protein C pathway and the tissue-factor-inhibitor pathway can be inhibited as well as fibrinolysis [6,7]. This leads to microvascular thrombosis and formation of intravascular fibrin and finally to multiple organ failure [8,9]. Beside the general response, a localized response to inflammatory agents can be seen depending on the type of tissue, tissue environment, cell type and endothelial cell response to inflammation [3,10,11]. Since organs differ in these factors, it is likely that local coagulation and fibrin deposition differ as well [8].

In bacterial inflammation, platelets are activated via thrombin and pro-inflammatory mediators [3], causing α-degranulation with an increase of P-Selectin expression on the platelet surface [12]. To assess platelet function, several functional assays have been suggested: capillary bleeding time (CBT), platelet function analysis (PFA) and impedance aggregometry (Multiplate®). PFA is commonly used to monitor anti-platelet therapy and as a screening tool for the von Willebrand disease [13]. Impedance aggregometry is used to monitor platelet function in patients with inherited platelet dysfunctions as well as aspirin®- and clopidogrel therapy to detect non-responders or anti-platelet drug resistance respectively [14-16]. Both tests are mainly based on platelet aggregation in solution induced by different platelet agonists. PFA but not impedance aggregometry assesses platelet adhesion and thrombus formation on platelet reactive surfaces. A distinction of platelet adhesion to collagen versus homotypic platelet-platelet interaction is not possible with any of the available platelet function assays. However, an exact diagnosis of disturbed thrombus formation on surfaces, especially in the setting of vessel wall injury and exposition of platelets to sub-endothelial structures like collagen, is important to prevent thrombotic events in the septic patient.

In a recent study we adapted methodological aspects to assess interactions between canine platelets and a collagen matrix in a flow chamber based adhesion assay and defined parameters that allow reproducible examination of thrombus formation under flow conditions in whole blood (Ferkau et al., in press). This system seems to be ideal to study the influence of inflammation on platelet adhesion and thrombus formation in comparison to conventional assays of platelet function such as capillary bleeding time, automatic platelet function analyses using the PFA-100® and impedance aggregometry using the Multiplate® impedance aggregometer.

The aim of the present study was to examine possible influences of naturally occurring bacterial infection and concomitant treatments on adhesion and thrombus formation of canine platelets as assessed by the flow chamber and to define the relationships between results obtained by flow chamber and conventional platelet function assays.

## Methods

### Study design

Blood samples of clinically healthy dogs (n = 16) and samples of dogs with bacterial inflammation (n = 8) were measured including platelet count and three conventional test procedures of primary hemostasis (in vivo capillary bleeding time, automatic platelet function analyzes using the PFA-100® and impedance aggregometry using the Multiplate® impedance aggregometer). Additional measurements were performed with the flow chamber using non-activated samples compared to samples with activated platelets using PAR 4 agonist. Supplementary in vitro experiments examined the influence of LPS (n = 5) and HES (n = 10) on the adhesion of platelets from healthy dogs.

### Animals

Dogs with bacterial inflammation were patients of the Small Animal Clinic, University of Veterinary Medicine Hannover. Included were patients presenting with a local or systemic infectious disease caused by bacteria (6 = abscess, 1 = peritonitis, 1 = hemorrhagic enteritis with sepsis) and systemic symptoms (body temperature 37.6–39.6°C, median 38.8°C; heart rate 96–134 beats per min, median 115 beats per min). Animals with infection had blood cell counts with platelet counts of 99,000–431,000/μL (median 269,000/μL), red blood cell counts of 4.2–9.1 × 10^6^/μL (median 5.9 × 10^6^/μL), white lood cell counts of 11.5–20.3 × 10^3^/μL (median 15.6 × 10^3^/μL) and hematocrit values of 27.0–57.0% (median 39.6%). Blood samples were drawn before treatment commenced.

Clinically healthy dogs used as controls were of different age, gender and breed. Health status was confirmed by a normal hematological and biochemical profile. Healthy animals had normal blood cell counts with platelet counts of 107,000–399,000/μL (median 251,000 /μL), red blood cell counts of 6.6–9.4 × 10^6^/μL (median 7.7 × 10^6^/μL), white blood cell counts of 5.5–10.2 × 10^3^/μL (median 7.5 × 10^3^/μL) and hematocrit values of 43.0–62.0% (median 52.0%).

Experiments were performed in accordance with the German Animal Welfare law and were approved by the official animal health care officer of the university and the local animal welfare authorities (Lower Saxony State Office for Consumer Protection and Food Safety).

### Blood sampling

Minimal pressure was deployed to raise the saphenous or cephalic vein by an assistant to facilitate venipuncture and blood draws. The samples were drawn using sterile disposable needles (18 G) into several plastic tubes. 3.8-mL tubes covered with buffered citrate solution (for PFA-100®, 0.129 mol/L trisodium citrate/citric acid buffer solution pH 5.5, Sarstedt AG & Co, Nuembrecht, Germany), 4.5-mL hirudin-coated tubes (Multiplate®, Verum Diagnostica GmbH, Munich, Germany) and 1.3-mL EDTA tubes (CBC) were used. Citrate anticoagulation (9 parts of blood to 1 of part 0.11 M citrate solution) was used for flow chamber studies. Blood and anticoagulant were immediately mixed carefully. The samples were stored at room temperature and used within 3 hours after blood collection.

Hematological analyses (platelet count, red blood cell count, hematocrit values) were measured automatically using the hematology system ADVIA 120 (Siemens Healthcare Diagnostics, Eschborn, Germany).

### Capillary bleeding time

For capillary bleeding time awake dogs were brought into lateral position. At the lateral side of the front toe the hair was removed with a shaver and hyperemia was induced (Finalgon® Crème, Boehringer Ingelheim, Germany). Close to the edge of the skin of the pad two punctures were placed with a sterile semi-automatic blood lancet (Softclix II, Roche Diagnostics, Mannheim, Germany). Before and during the measurement a steady pressure of 70 mmHg was maintained with a blood pressure cuff. Every 15 seconds blood drops were dabbed off carefully until the bleeding stopped. The average bleeding time was calculated as the mean time of the two punctures [17].

### Platelet function measurements with PFA 100®

Measurements with the platelet function analyzer PFA-100® were performed according to the manufacturer’s instructions using the collagen/adenosine-5′-diphosphate (CAPD) cartridge and the collagen/epinephrine (CEPI) cartridge (Siemens Healthcare Diagnostics GmbH, Eschborn, Germany). The blood sample was filled into the cartridge and sucked under a constant vacuum of 40 mmHg through a capillary membrane. The membranes central aperture is coated with different agonists that induce platelet activation. “Closure time” is defined as the time, which is required to completely close this aperture (in sec, maximal testing time 300 sec). The “total volume” (in μL) describes the amount of blood needed to achieve closure.

### Platelet aggregation in Multiplate® impedance aggregometer

Impedance aggregometry was performed with the Multiplate® impedance aggregometer according to the manufacturers recommendations.

300 μL blood were mixed gently with 300 μL 0.9% NaCl and incubated for 3 minutes at 37°C before 20 μL of the respective agonist solution were added. Thrombus formation was followed for 12 minutes. Multiplate® detects an increase in electrical impedance caused by platelets attaching to the surface of the sensor wires. Results are converted by the built-in algorithm into arbitrary “Aggregation Units” (AU). The “Area under the curve” (AUC in AU*min) is an integrated measure of the velocity (AU/min) of aggregation and is reported to reflect maximal aggregation. Agonist concentrations were 5 μg/mL collagen, 10 μmol/L adenosine diphosphate and 1 mmol/L arachidonic acid [18]. Instruments and reagents were purchased from Verum Diagnostica GmbH (Munich, Germany).

### Measurements in the flow chamber

#### Substrate coating

To prepare a canine collagen solution 10 mg of lyophilized canine collagen (YO Proteins AB, Huddinge, Sweden) were diluted in 10 mL of 2.5 mol/L acetic acid and kept on a tumbler for 5 hours at 4°C. Subsequently the remaining sediment was resolved by a homogenizer. The stock solution was diluted with Dulbecco’s phosphate buffered saline (DPBS, Lonza, Cologne, Germany) to achieve a final concentration of 200 μg/mL canine collagen.

The substrate is a plastic chip which can be coated with matrix proteins like collagen. Before coating substrates were washed in 70% ethanol and in DPBS. 125 μL of the collagen solution was placed in a Petri dish and the substrate carefully placed on it. The substrate was incubated at least 2 hours in the refrigerator. Before use the substrate was gently washed in DPBS to remove excess collagen solution.

#### Platelet staining

Platelets in whole blood were stained with the fluorescent dye Dihexylcarbocyanine Iodide (DiOC_6_, Invitrogen, Frankfurt, Germany) at a final concentration of 1 μmol/L. To achieve this, 10 μL of the stock solution (prepared to manufacturer’s instructions) were added to 1 mL of citrated blood and incubated for 30 minutes under exclusion of light. The success of the staining was controlled microscopically in a native preparation.

#### PAR 4 agonist preparation

The stock solution of the agonist PAR 4 was prepared by dissolving the lyophilized reagent in 1 mL purified water to achieve a concentration of 20 mmol/L. Aliquots of 50 μL were produced and according to recommendations of the manufacturer stored at −20°C for a maximum of 4 weeks.

#### Flow chamber

The flow chamber based adhesion assay using citrated whole blood was performed in a disposable biochip perfusion chamber (VenaEC™, Cellix Ltd., Dublin, Ireland). The biochip perfusion chamber only requires approximately 100 μL of blood for a 3 minute experiment at a wall shear stress of 14 dynes/cm^2^. The biochip was developed for human samples and mimics human capillaries. The biochip was placed in DPBS for at least 30 minutes according to recommendations of the manufacturer to soak the silicone membranes of the channel to avoid leakiness of the channel when mounting it on the substrate. Each biochip consists of 2 parallel channels (flow chamber dimensions: 20 mm length × 600 μm width × 120 μm depth) and is placed on the associated collagen coated substrate. The cannels are measured consecutively.

To accomplish a wall shear stress of 14 dynes/cm^2^ during the experiment, a precision microfluidic syringe pump (Mirus™ 2.0 Nanopump, Cellix Ltd., Dublin, Ireland) was used which was controlled by a personal computer in combination with the recommended software (FlowAssay™ Software, Cellix Ltd., Dublin, Ireland). The pump was employed in withdrawal mode to generate a flow that was calculated to achieve the preset shear rate.

#### Test procedure

At the beginning of the experiment, the biochip was mounted on the collagen coated substrate and placed on the stage of an inverted epifluorescence microscope (Olympus, Hamburg, Germany) equipped with a CCD camera (Retiga EXi, Qimaging, Surrey, Canada). The measurement channel was rinsed with Hank’s Balanced Salt Solution (HBSS, Lonza, Cologne, Germany). Subsequently stained whole blood was perfused through the channel for 3 minutes. Afterwards the channel was washed out with HBSS for 1 minute before 10 pictures of 115,6 mm^2^ (500 × 500 frames) each on the entire length of the passage were recorded at 40-fold magnification. Pictures were recorded still under flow conditions.

To achieve platelet activation 100 μL samples of whole stained blood were incubated with 10 and 20 μL PAR 4 agonist to achieve a final concentration of 1.8 mmol/L. Platelets were incubated for 10 minutes at room temperature and immediately perfused into the biochip.

#### Measurement parameters

Areas of interest (parameter threshold) were defined manually in every single picture to detect all districts covered by fluorescing platelets. For one channel 10 corresponding pictures were measured. Platelet covered area and average thrombus sizes were calculated in every picture and a mean was calculated for each parameter per channel.

Software "groß" ImageJ 1.38× (National Institutes of Health, USA) was used to measure the total area covered by adhering platelets (μm^2^) and the average size of individual thrombi (μm^2^). The detection limit was set to > 4 μm^2^ (corresponding to at least 2 platelets).

### In vitro experiments

To investigate the influence of LPS, blood samples from healthy dogs were incubated for 30 minutes with 0.3 ng/mL LPS (*E. coli*, serotype 0111:B4, Sigma Aldrich, Taufkirchen, Germany).

The influence of HES on platelet function was investigated by incubating 100 μL blood samples of healthy dogs with 12 μL of HES 6% (Vitafusal® 6%, 130 kDA, Serumwerk Bernburg AG, Bernburg, Germany) for 10 minutes prior to perfusion experiments. The dose was chosen based on the recommended dose for patients receiving a dose of 10 mL HES per kg body weight.

### Statistical analysis

Data were tested for normal distribution using the Kolmogorov-Smirnov test. Data were normally distributed for all data families except for the HES study. Statistical significance was thus assessed with t-tests for normally distributed data (paired and unpaired t-tests) and with non-parametric tests for the non-normally distributed data of HES co-incubation (Wilcoxon test for paired data, Mann–Whitney U test for unpaired data). These data are presented as medians and 95% CI and graphically depicted as box plots. Areas under the receiver operating characteristics curve (AUC of ROC) were compared as described by DeLong [19]. The compared AUCs contained non-nested data [20]. Two-sided values of *P* < 0.05 were considered as significant.

## Results

### Hematocrit and red blood cell count

Hematocrit values and red blood cell counts in dogs with inflammation were significantly decreased in comparison to healthy dogs (*P* < 0.05, unpaired t-test).

### Capillary bleeding time

Capillary bleeding time (25/75% percentile; healthy dogs 58–92 sec, median 71 sec vs. dogs with inflammation 37–123; median 84 sec) in healthy dogs and dogs with inflammation showed no significant difference (*P* > 0.05, unpaired t-test).

### Platelet function analyzer

**Figure 1:**
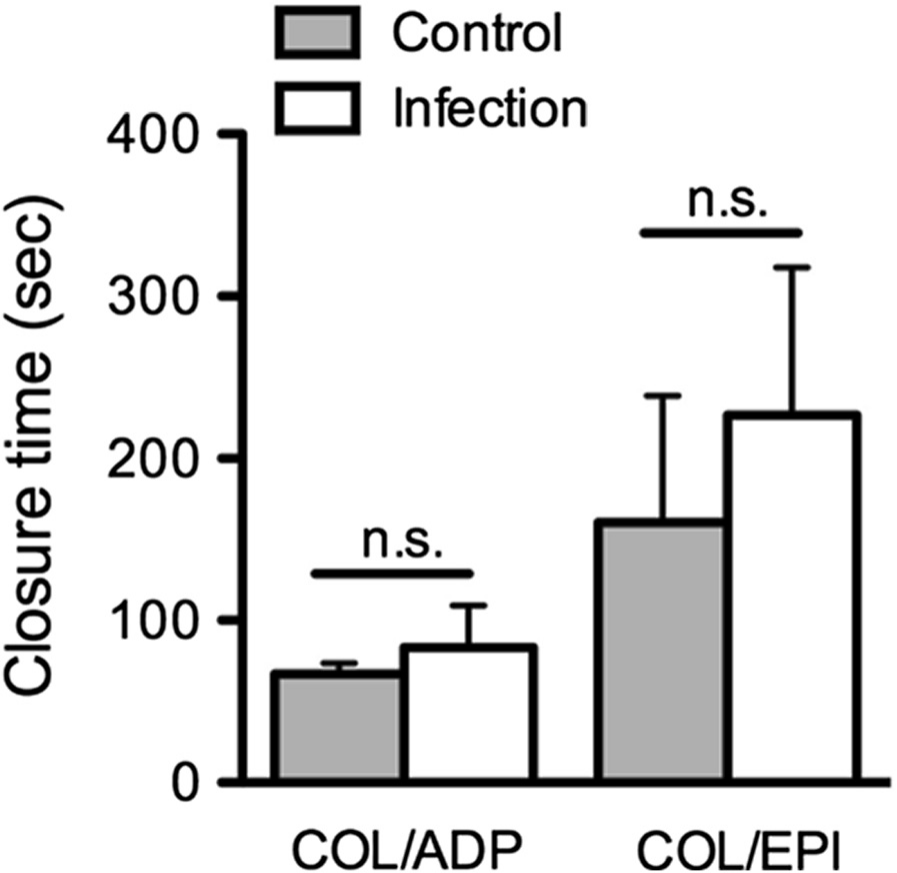
**Platelet dysfunction in dogs with infection as assessed in the platelet function analyzer (PFA).** Closure time of dogs with bacterial inflammation (n = 8) in comparison to clinically healthy dogs (n = 16) measured in the platelet function analyzer with the cartridge types collagen/adenosine-5′-diphosphate (COL/ADP) and collagen/epinephrine (COL/EPI) (mean ± SD; *P* > 0.05, unpaired t-test).

While a trend was seen with respect to a reduced collagen-mediated activatability did the PFA parameters “closure time” and “total volume” not show significant differences when comparing healthy dogs and dogs with infection (Figure 1, closure time; total volume COL/ADP: 252; 231/275 μL vs. 286; 232/360 μL; total volume COL/EPI: 392; 279/737 μL vs. 638; 257/871 μL; *P* > 0.05, unpaired t-test).

### Impedance aggregometry

**Figure 2:**
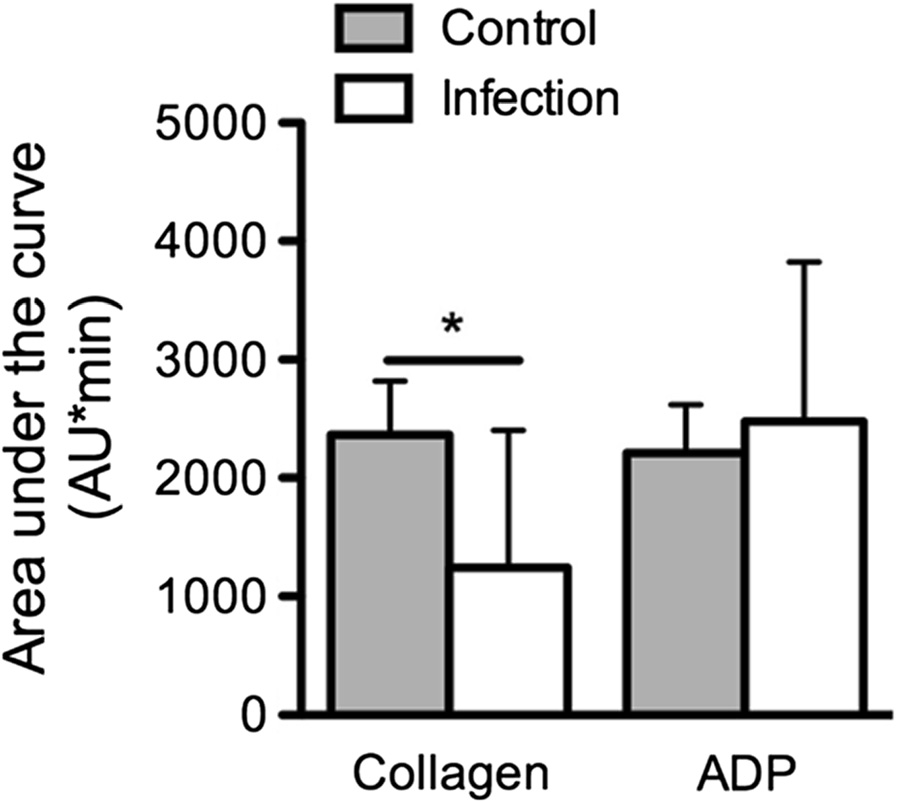
**Platelet dysfunction in dogs with infection as assessed in impedance aggregometry.** Area under the curve (AU*min) of dogs with bacterial inflammation (n = 8) in comparison to clinically healthy dogs (n = 16) measured in the impedance aggregometry with the agonists collagen and ADP (mean ± SD; * = *P* < 0.05, unpaired t-test).

In contrast, in impedance aggregometry platelet aggregation of dogs with infection was significantly decreased in comparison to healthy dogs when using the agonist collagen (*P* < 0.01, U-test) (Figure 2) but not with ADP. (AUC values of arachidonic acid: 1686; 1004/2874 AU*min vs. 867; 266/3919 AU*min).

### Results of flow chamber measurements

**Figure 3:**
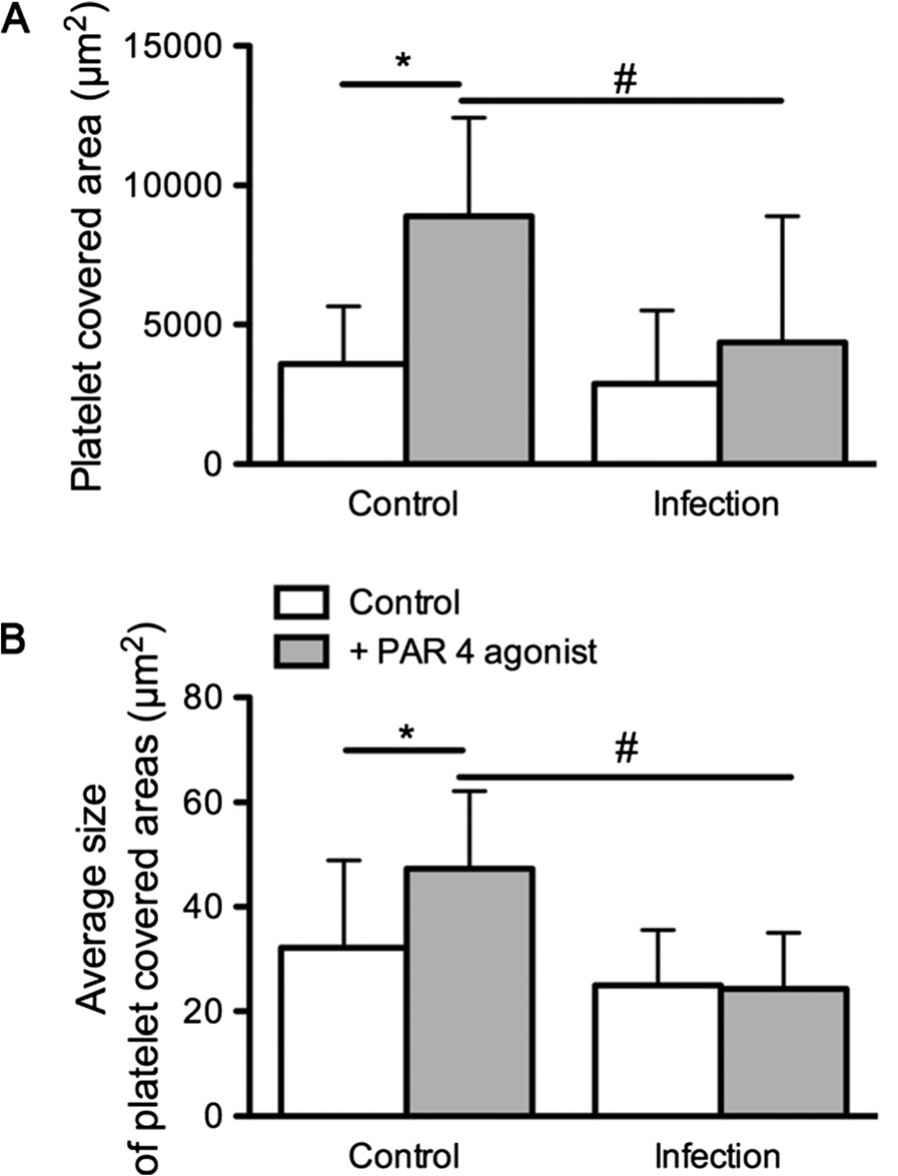
**Platelet dysfunction in dogs with infection as assessed in the flow chamber.** Platelet covered area **a**) and average size of platelet covered areas **b**) of dogs with bacterial inflammation (n = 8) in comparison to clinically healthy dogs (n = 16) investigated in the flow chamber under standardized conditions (canine collagen 200 μg/mL, wall shear stress 14 dynes/cm^2^) in non-activated and PAR 4 agonist activated platelets (mean ± SD; * = *P* < 0.05; paired t-test, # = *P* < 0.05; unpaired t-test).

Platelet adhesion to collagen and thrombus formation in the flow chamber did not show significant differences in platelet covered area and average size of platelet covered areas of non-activated platelets of healthy dogs and dogs with infection (*P* > 0.05, unpaired t-test). Platelet covered area (*P* < 0.01, paired t-test) and average size of platelet covered areas (*P* < 0.01, paired t-test) of healthy dogs increased significantly after platelet activation with PAR 4 agonist. In contrast, in dogs with infection neither platelet covered area nor average size of platelet covered areas increased after platelet activation with PAR 4 agonist (*P* > 0.05, paired t-test). After platelet activation with Bindestrich weg, platelet adhesion and average size of platelet covered areas in dogs with infection was significantly decreased in comparison to healthy dogs (unpaired t-test, *P* < 0.05) (Figure 3).

### Results of areas under the ROC curve

We next asked, which of the examined platelet function parameters would detect infection-associated platelet dysfunction with the highest sensitivity. Areas under the ROC curve were 0.54 (CBT), 0.66 (PFA COL/ADP) and 0.57 (Multiplate® impedance aggregometry; agonist collagen). For flow chamber studies area under the ROC curve was 0.91 for average size of platelet covered area after platelet activation with PAR 4 agonist.

**Figure 4:**
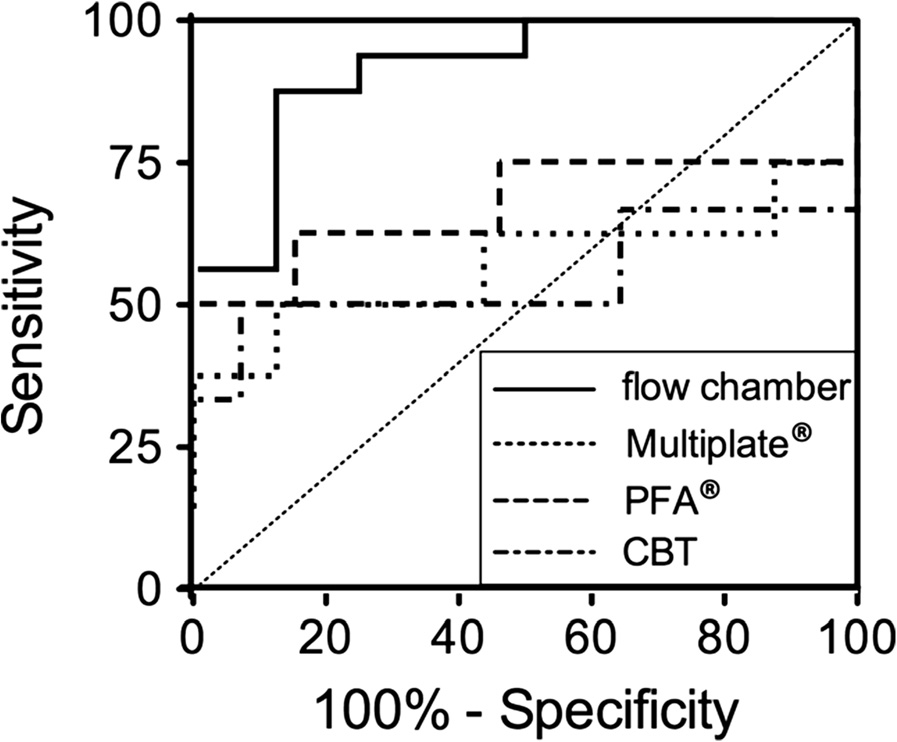
**Receiver operating curve analysis of different assays of platelet function.** Comparison of areas under the receiver operating characteristics curve (AUC of ROC) of CBT, PFA COL/ADP, impedance aggregometry (agonist collagen) and the parameter average size of platelet covered area after activation with PAR 4 agonist. Two-sided values of *P* < 0.05 were considered as significant.

The AUC of average size of platelet covered area was significantly higher than the AUC of Multiplate® impedance aggregometry (*P* < 0.05, DeLong test) (Figure 4). The sensitivities and specificities of the tests that were compared in the ROC analysis are displayed in Table 1.

**Table 1:** Sensitivities and specificities of tests compared in the ROC analyses

**Parameter**	**Criterion**	**AUC**	**Sens**	**Spez**
FC Size + PAR	≤ 30,76	0,914	87,5	87,5
MP Coll AUC	> 2713	0,570	50,0	87,5
PFA Coll/ADP VZ	> 81	0,663	50,0	100,0
CBT	> 97,5	0,542	50,0	92,9

Flow chamber derived parameters could deliver means to improve sensitivity and specificity of tests batteries for the detection of relevant platelet dysfunction compared to the use of established methods to assess platelet function.

### In vitro addition of LPS

**Figure 5:**
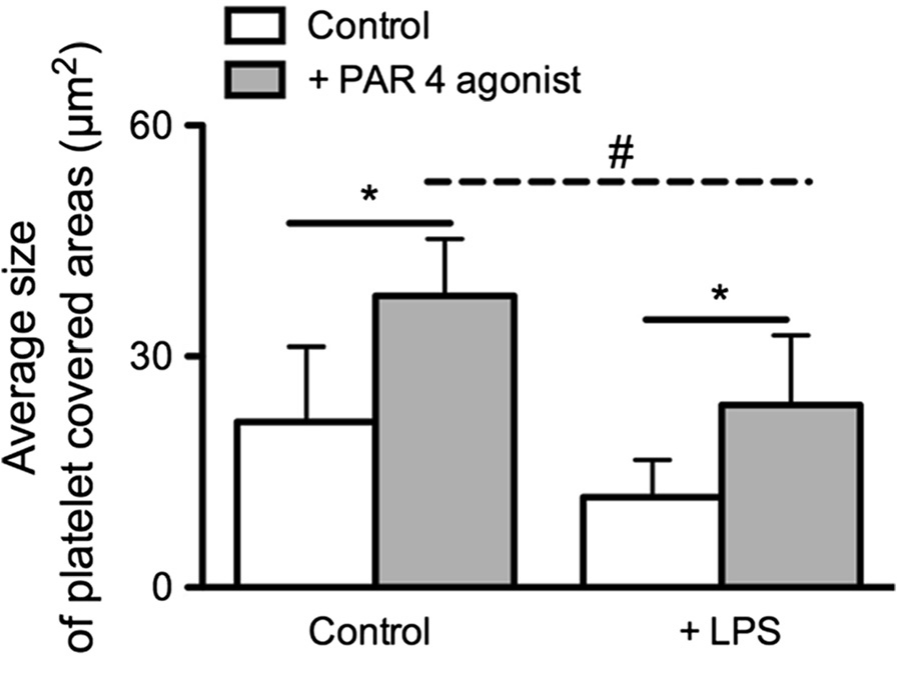
**LPS-induced platelet dysfunction as assessed by the flow chamber.** Average size of platelet covered areas of samples treated with LPS (n = 5) in comparison to non-LPS-treated samples investigated in the flow chamber under standardized conditions (canine collagen 200 μg/mL, wall shear stress 14 dynes/cm^2^) in non-activated and PAR 4 agonist activated platelets (mean ± SD; * = *P* < 0.05, paired t-test; # = *P* < 0.05, unpaired t-test).

Bacterial wall components like LPS have been shown to induce sepsis-associated platelet dysfunction. To assess if LPS-induced platelet dysfunction can be detected in the flow chamber we conducted additional in vitro studies. After in vitro addition of LPS to examine the influence of endotoxin on platelet function, average size of platelet covered areas (*P* < 0.05, unpaired t-test) of PAR 4 agonist activated platelets were significantly lower in activated LPS treated samples than in the activated non-treated control. As in the control, average size of platelet covered areas (*P* < 0.05, paired t-test) increased significantly after platelet activation with PAR 4 agonist in LPS treated samples (Figure 5).

### In vitro additon of HES

**Figure 6:**
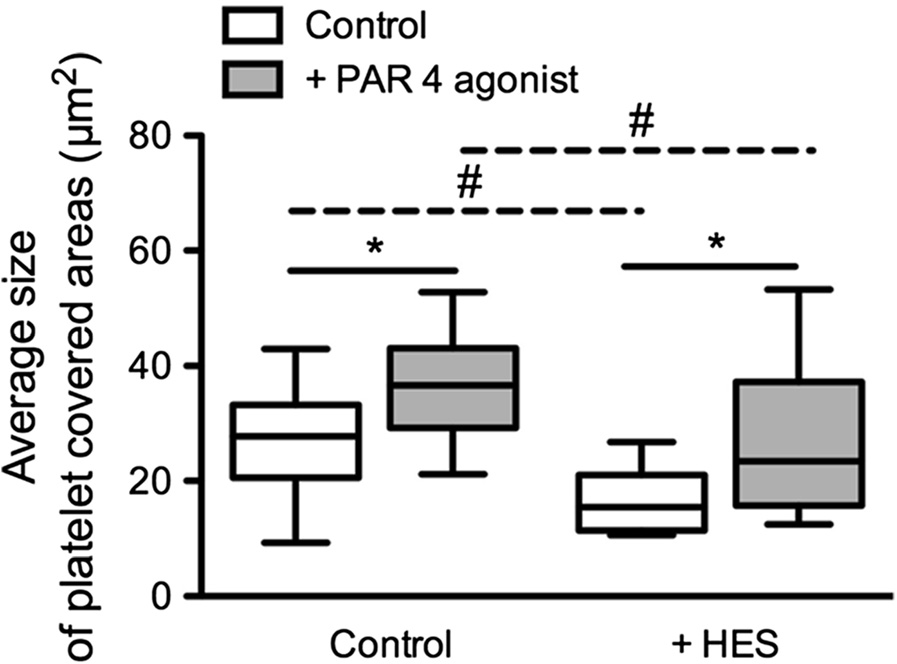
**HES-induced platelet dysfunction as assessed in the flow chamber.** Average size of platelet covered areas of samples treated with HES (n = 10) in comparison to non-HES-treated samples in the flow chamber under standardized conditions (canine collagen 200 μg/mL, wall shear stress 14 dynes/cm^2^) in non-activated and PAR 4 agonist activated platelets (median ± min/max; * = *P* < 0.05, Mann–Whitney U test; # = *P* < 0.05, Wilcoxon test).

Therapy of infection with systemic symptoms mainly relies on antibiotic treatment and replacement of volume depletion. Since colloids, i.e. starch has been demonstrated to induce platelet dysfunctions, we tested in vitro if the flow chamber would be affected by clinically used treatment strategies. In vitro addition of HES caused a significantly decreased average size of platelet covered areas (*P* < 0.05, Mann–Whitney U test) in comparison to the non-treated control. As in the control, platelet activation with PAR 4 agonist in the HES treated samples increased average size of platelet covered areas (*P* < 0.05, Wilcoxon test). Average size of platelet covered areas after activation was significantly lower in comparison to average size of platelet covered areas after activation in the non-treated control (*P* < 0.05, Mann–Whitney U test) (Figure 6).

## Discussion

The present study demonstrates that conventional assays of platelet function provide limited sensitivity to detect platelet dysfunctions in dogs associated with infection. In contrast, the flow chamber detects infection associated platelet dysfunction in dogs, since platelet activation in response to PAR 4 agonist was disturbed in these animals. In vitro, addition of LPS highlights the inhibiting effect of LPS on platelet function. Platelet dysfunctions, which are associated with the substitution of HES, are as well represented in the flow chamber.

In contrast to other platelet function assays, capillary bleeding time and platelet function analyzer did not detect significant differences in healthy dogs and dogs with infection. This was also confirmed by the calculated AUCs of the ROC. The various assays for platelet function used in this study are known to detect different aspects of platelet function. Bleeding time is a global coagulation parameter, closely resembling the clinical setting but it is regretfully not very specific or sensitive for any bleeding disorder, because results depend on factors of the animals (thickness of the skin, blood pressure, vessel-wall structure) and on technical factors (intensity of hyperemia, infliction of the puncture lesion) as well as on breeds and individuals [21,22].

As already mentioned, platelet function analyzer is normally used to detect anti-coagulant substances in patients. Platelet function is measured under non-physiological, high wall shear stress conditions from 150 up to 190 dynes/cm^2^[23,24]. It is known that already a wall shear stress from up to 50 dynes/cm^2^ leads to changes in platelet morphology, granule secretion and platelet aggregation [25]. Changes in platelet function caused by a high wall shear stress may mask a mildly disturbed platelet function induced by infection. PFA does however incorporate a platelet-wall interaction component into the analysis. In addition, platelet count and hematocrit values have to be considered since both values influence the test result of the platelet function analyzer [26,27].

The AUC of the ROC of the Multiplate® impedance aggregometer, at least using the agonist collagen, indicated that the Multiplate® impedance aggregometer detected platelet dysfunction induced by infection. In contrast to the platelet function analyzer, impedance aggregometry measures platelet aggregation in response to platelet agonists under conditions of low shear stress but without a distinct vessel wall component. For the experiment, blood samples are diluted in normal saline [18]. Nevertheless, platelets of dogs with infection showed a significantly attenuated aggregation response to collagen. This result indicates reduced platelet function and a reduced platelet response to this matrix protein in dogs with infection. Interestingly was the activation in response to ADP not affected.

In our flow chamber studies, platelet covered area and average size of platelet covered areas in dogs with infection and healthy dogs did not show significant differences at baseline. However, platelets of dogs with infection failed to respond to platelet activation with PAR 4 agonist. That is particularly interesting since in impedance aggregometry the TRAP test is considered as a positive control that remains unaffected by most dysfunctional states of platelets.

In inflammation, circulating mononuclear cells express tissue factor, which sets off the plasmatic coagulation cascade and ultimately generates thrombin from pro-thrombin. Thrombin is a potent agonist to activate platelets via protease activated receptors [28] and leads to platelet shape change, granule secretion and platelet aggregation [29-31]. An increased expression of P-selectin was detected on the platelet surface of dogs with inflammatory diseases, which indicates an in vivo activation and degranulation [12]. In other studies “platelet exhaustion” was detected in dogs with pancreatitis [32]. This exhaustion is viewed as the consequence of submaximal activation after platelet exposure to thrombin in vivo secondary to the generation of immune complexes or contact to damaged endothelium [33].

Platelet exhaustion can also be the cause for the lack of responsiveness to PAR 4 agonist activation of platelets in dogs with infection. Changes in receptor expression on the platelet surface are probably not the reason for missing platelet activation, as receptor expression and adhesion molecules remain unchanged in human patients with sepsis [34]. Nevertheless, does the missing response of platelets to PAR 4 agonist activation allow discrimination between healthy dogs and dogs with infection in the flow chamber.

This is also demonstrated by the AUCs of the ROC of the flow chamber parameters, since both platelet covered area AUC of the ROC and average size of platelet covered area AUC of the ROC for the PAR 4 agonist condition provide very good prediction of the presence of platelet dysfunction secondary to infection.

The comparison of the AUC of ROC of Multiplate® impedance aggregometry and flow chamber parameters after platelet activation with PAR 4 agonist indicates a trend to an improved sensitivity of the flow chamber, probably due to a better representation of the in vivo situation in the flow chamber. This trend is also corroborated by the higher specificity and sensitivity of flow chamber parameters compared to the standard tools to assess platelet function.

Hematocrit values were significantly decreased in dogs with infection. To eliminate the possibility that hematocrit values affect platelet response to agonists and to highlight the situation of infection, blood samples of healthy dogs with normal hematocrit values were incubated with LPS and HES. After in vitro addition of LPS there was no significant difference between the control and LPS treated samples under resting conditions. Interestingly, platelet activation with PAR 4 agonist still increased average size of platelet covered area. In comparison to the non-LPS-treated control average size of platelet covered area after platelet activation with PAR 4 agonist in LPS treated samples was significantly decreased but responsiveness was not completely abolished. We chose LPS because an inhibitory effect of LPS on platelet aggregation has previously been described by Saba et al. [35] in platelet rich plasma. Most of our patients presented with abscesses that are frequently caused by staphylococci and not by gram-negative E. coli. We used LPS as one example for bacterial wall components. Similar studies with lipoteichoic acid or other bacterial activators would likely yield comparable effects. This inhibitory effect of LPS on platelet responsiveness may have been induced by direct binding of LPS to the platelet surface [36]. LPS altered platelet responses to agonists in a study of Nystrom et al. [37], but it has to be considered that the effect of LPS on platelets is dose dependent [38]. In the present study we used LPS at a concentration of 0.3 ng/mL, which is a concentration that has been observed in patients with sepsis [39]. Since Nystrom et al. [37] used a much higher concentration of 80 mg/L, which probably does not reflect the situation in vivo, it is not surprising that results of the two studies differ.

Another potential reason for disturbed platelet responsiveness to thrombin activation could be infusion therapy in septic patients. In order to investigate if the first line therapy would affect the performance of the flow chamber experiments, we substituted HES in blood of healthy animals. In vitro substitution of HES to blood samples of clinically healthy dogs in an equivalent dose to 10 mL/kg body weight decreased average size of platelet covered areas. Platelet activation with PAR 4 agonist increased average size of platelet covered areas in HES treated samples and in the control, but in comparison to the control average size of platelet covered area in HES treated samples was significantly reduced. The resulting picture does however not resemble the one caused by clinically significant infection. An inhibiting effect of HES on platelet function and blood coagulation has previously been described. For example after substitution of HES in dogs a prolonged platelet closure time in the PFA was seen, but only at a dose of 20 mL/kg body weight [40,41]. A dose dependent effect of HES was described in humans by Liu et al. [42], who also detected notable effects on platelet function after greater than 20% dilution with HES. A possible inhibitory mechanism of HES on platelet function is an unspecific coating of platelets by HES macromolecules [43], which may decrease the availability of the platelet glycoprotein IIb-IIIa complex [44]. Our results indicate an inhibiting effect of HES on platelet function, even at a low concentration of substitution. In contrast, a low dose of HES does not affect the possibility of platelet activation. Taken together, substitution of HES in a low dose probably only leads to a slight hemodilution, but may worsen an already altered hemostasis in patients with inflammation. The effect of HES infusions alone does however not explain the total lack of the platelet response seen in dogs with infection. HES therapy would however not mask the diagnosis of sepsis associated platelet dysfunction and render flow chamber studies to secure this diagnosis useful.

## Conclusion

The flow chamber was able to detect infection associated platelet dysfunction in dogs, by unmasking a lack of a platelet response to PAR 4 agonist. Our small proof of principle study should be followed up by a larger prospective study that addresses the question if platelet dysfunction is useful to detect infection, if infection-associated platelet dysfunction detected by the flow chamber is associated with clinically relevant bleeding and if flow chamber parameters are indeed superior to conventional parameters like PFA and Multiplate® to detect this dysfunction.

## Competing interests

The authors declare that they have no competing interests.

## Authors’ contributions

AF adapted methodological aspects of the method, carried out flow chamber studies and drafted the manuscript. HJG drafted the manuscript and its revisions and performed the statistical analysis. RM provided the animals used in the study and participated in the study design and coordination. SC and SE helped to perform flow chamber studies. MA and JWM carried out platelet function analysis and impedance aggregometry. GT conceived of the study, and participated in its design and coordination and helped to draft the manuscript. All authors read and approved the final manuscript.
